# Research on the early warning indicator system for red tide disasters in the nearshore sea area in Qinhuangdao

**DOI:** 10.1038/s41598-026-40344-6

**Published:** 2026-02-28

**Authors:** Liu Yu, Wu Yuchen, Zhang Ning, Wang Xueying, Li Yan, Jing Guoning, Tian Zhen

**Affiliations:** 1 National Ocean Technology Center, Tianjin, 300112 China; 2 College of Fisheries, Tianjin Agricultural University, Tianjin, 300384 China

**Keywords:** Red tides, Early warning, Indicators, Climate sciences, Environmental sciences, Natural hazards, Ocean sciences

## Abstract

In order to construct an early warning indicator system for red tide disasters in the nearshore sea area of Haigang District in Qinhuangdao, this study analyzes data collected from online buoy monitoring from April to October 2024, including temperature, salinity, chlorophyll-a, pH, turbidity, nitrate, and dissolved oxygen (DO) data, along with red tide information from monitoring based on satellite remote sensing. The results indicated that during the monitoring period, red tides occurred 16 times, with a cumulative duration of 55 days and a total affected area of 10,047 km^2^. The typical duration and affected area of a single red tide ranged from 1 to 2 days and 195 to 850 km^2^, respectively. The occurrence of red tides in the nearshore sea area of Haigang District is associated with environmental factors such as chlorophyll-a, nitrate, DO and its range, among others. Taking each environmental factor as an early warning indicator for red tides, both chlorophyll-a and nitrate showed an increasing trend in missing alarm rate (MAR) with the increase in the red tide incidence. The study results showed that red tides in the nearshore sea area of Haigang District present the characteristics of high frequency, long durations, and large average areas from late summer to early autumn (August to October). The DO range and nitrate are reliable early warning factors of red tide occurrence in the sea area.

## Introduction

Red tides, also known as harmful algal blooms (HAB), refer to an ecological anomaly characterized by the rapid proliferation or aggregation of certain micro-phytoplankton, protozoa, or bacteria in a body of water under specific environmental conditions, resulting in a change in the color of the water body^1^. This phenomenon causes severe damage to the ecological environment of the sea area concerned and poses threats to human health and local economic development^2^. Research has shown that in northern China, climatic influences have led to an increase in sea surface temperatures, resulting in a rising trend in both the duration and affected area of red tides^3,4^. Time-series analyses conducted by some researchers have indicated that the frequency of red tide occurrences is on the rise^5^.

The formation of red tides is closely associated with multiple environmental factors, especially eutrophication of water bodies, which serves as the primary driver. Research has shown^2^ that red tide-prone areas are typically concentrated in sea areas with elevated nutrient concentrations, with a significant correlation between the increase in nitrogen and phosphorus nutrients and the occurrence of red tides. Chlorophyll-a, as the main pigment for phytoplankton photosynthesis, is often used to indicate the biomass of red tides. Researchers have studied^6–19^ the relationships between chlorophyll and factors such as DO, temperature, salinity, and pH. They found that chlorophyll is significantly negatively correlated with DO and salinity, while showing a significant positive correlation with pH; and red tides are also associated with nutrients and meteorological factors^20–23^. Currently, there are three monitoring methods for early warning of red tides: traditional monitoring, satellite monitoring, and online monitoring^6–8^. Traditional monitoring relies on vessel and manual monitoring of red tides for biological/chemical analyses of water body samples. Satellite monitoring uses satellite remote sensing^7^ to obtain information such as water color, allowing direct monitoring of dynamic changes in red tides, but it is costly and susceptible to weather conditions. Online monitoring, achieved through the deployment of buoys^8^, enables continuous real-time automatic monitoring of marine environmental factors, tracking and monitoring the entire process from the occurrence to the decline of red tides. This method is crucial for researching early warning mechanisms for red tides and achieving rapid alerts.

The nearshore sea area of Haigang District is home to important coastal ecosystems, such as wetlands, and is rich in marine biological resources, holding significant ecological and economic value. In recent years, with the intensification of global warming and marine pollution, the frequency, scale, and affected range of red tides in these sea areas have shown an upward trend^16,20^. This study utilizes online buoy monitoring to obtain dynamic changes in various indicators during red tide events in the sea area. Through statistical analysis of the red tide early warning monitoring data for the sea area, it aims to provide data support for the establishment of a red tide early warning system.

## Materials and methods

### Data sources

**Fig. 1:**
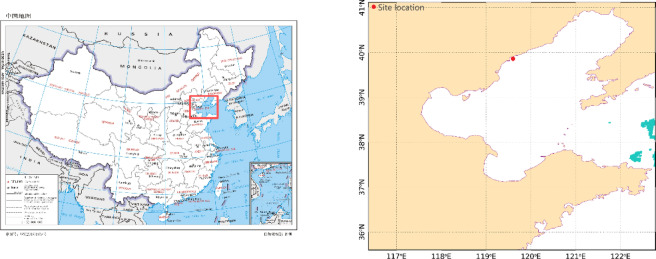
Schematic diagram of red tide monitoring buoy stations in Beidaihe, Qinhuangdao.

The data used in this study comprises marine environmental information and red tide data from the nearshore sea area of Haigang District in Qinhuangdao. Specifically, the marine environmental information is sourced from the results of monitoring using online buoys (Fig. 1) deployed in the study area, which includes chlorophyll-a, turbidity, water temperature, salinity, pH, DO, and nitrate. The buoys are multi-parameter water quality monitoring instruments independently developed by the National Ocean Technology Center, with data collection occurring hourly from April 26 to October 30, 2024. Due to equipment maintenance and data calibration during the period, there were gaps in the data: DO and nitrate data from May 10 to June 5, pH, nitrate, and turbidity data from June 5 to July 18, and all data from July 18 to August 16 and October 12 to October 13 were missing. Red tide information was derived from water color remote sensing data acquired by four satellites (AUQA, TERRA, JPSS1, and SNPP), including remote sensing reflectance and chlorophyll-a concentrations. Fuzzy C-Means (FCM) clustering analysis was used, utilizing the slopes between spectral bands of the remote sensing reflectance curve as a feature to determine the location and area of red tide occurrences, thereby acquiring red tide information from buoy monitoring points. To validate the reliability of the red tide determination results based on satellite remote sensing data, random field surveys were conducted during the study period.

### Methods

Based on data availability, this study selected temperature, salinity, chlorophyll-a, turbidity, DO, DO range, pH, and nitrate as environmental factors. The levels of these factors were quantified at red tide incidences of 100%, 90%, 80%, 70%, 60%, and 50%, supplemented by the missing alarm rate (MAR) to screen the environmental factors and their levels in the early warning of red tides. The formulas for calculating the red tide occurrence accuracy rate and MAR are as follows:

$$Occurrence\;Accuracy\;Rate = \frac{TP}{{TP + FP}}$$

$$MAR = \frac{FN}{{TP + FN}}$$

where, TP is the number of days when red tide events were detected by satellite remote sensing; FP is the number of days when online buoy monitoring data fell within the monitoring indicator range for red tide events, but satellite remote sensing did not detect the number of days for red tide events; FN is the number of days when buoy monitoring results did not meet the red tide occurrence indicator range criteria, but the satellite remote sensing detected the number of days for red tide events.

Data processing was completed using Excel 2021 and R3.6.2 software.

## Results

### Red tide characteristics

**Fig. 2:**
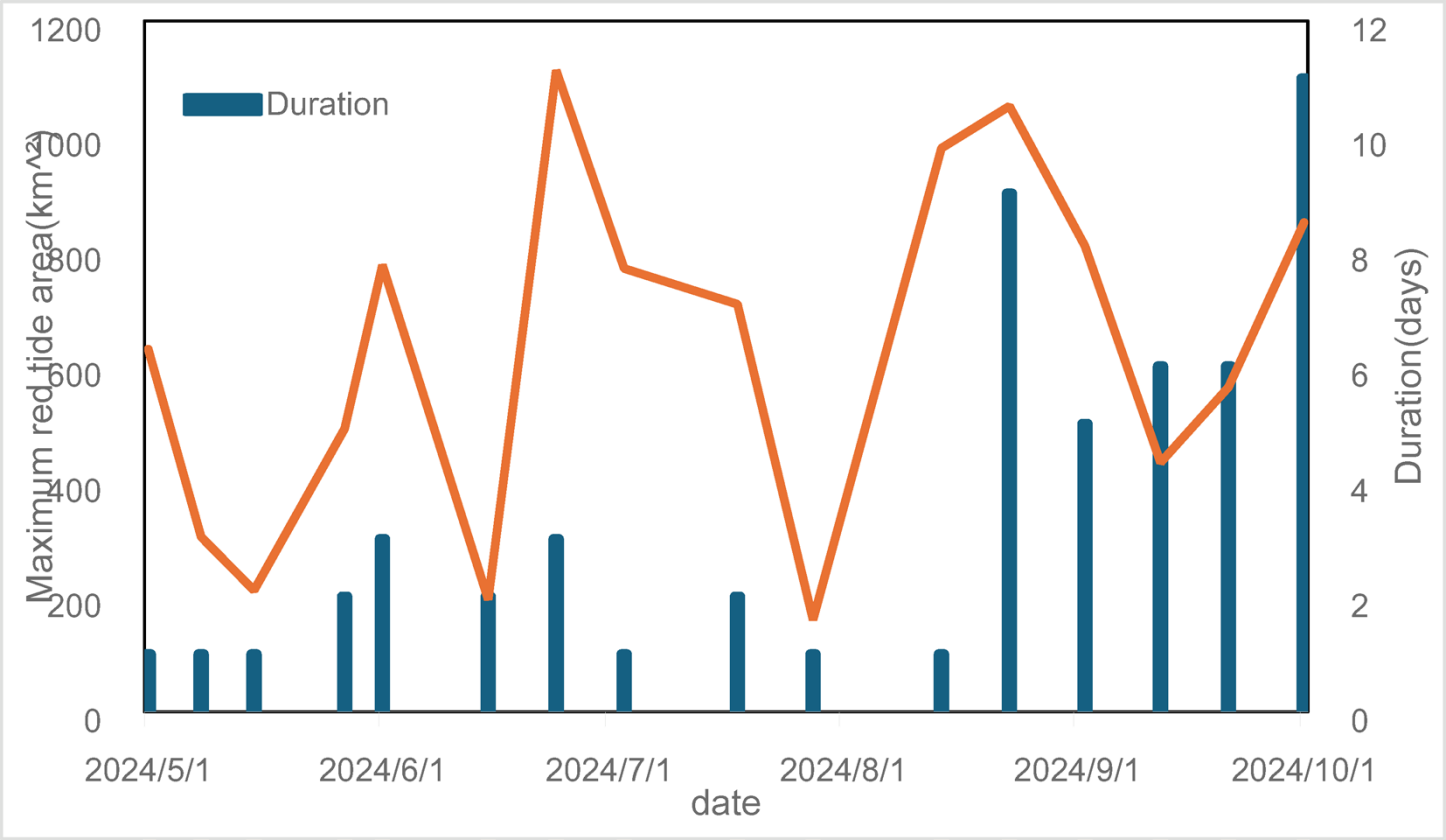
Duration and affected area of red tide events.

Satellite monitoring data (Fig. 2) indicate that a total of 16 red tide events were recorded in the nearshore sea area of Haigang District, with a total duration of 55 days and a cumulative affected area of 10,047 km^2^. Among months with recorded red tides, May had the highest frequency, with 4 occurrences, while October had the lowest, with only 1 occurrence. Notably, no events were recorded in April. The duration of a single red tide ranged from 1 to 11 days, with an average duration of 3.4 days. From May to July, red tide occurrences primarily lasted 1 to 2 days, accounting for 80% of the total occurrences during this period. In contrast, from August to October, the durations were generally longer; except for one red tide on August 14 that lasted 1 day, the others lasted 5 days or more, with the red tide on October 1 lasting 11 days. The average area affected by red tides was 627.9 km^2^. Excluding the maximum red tide area of 1114 km^2^ that occurred from June 24 to 26, the average area from May to July was 471.8 km^2^, while from August to October, the average area increased to 781.2 km^2^, with August’s average area reaching the highest monthly value of 1015.5 km^2^.

### Characteristics of environmental changes

**Fig. 3:**
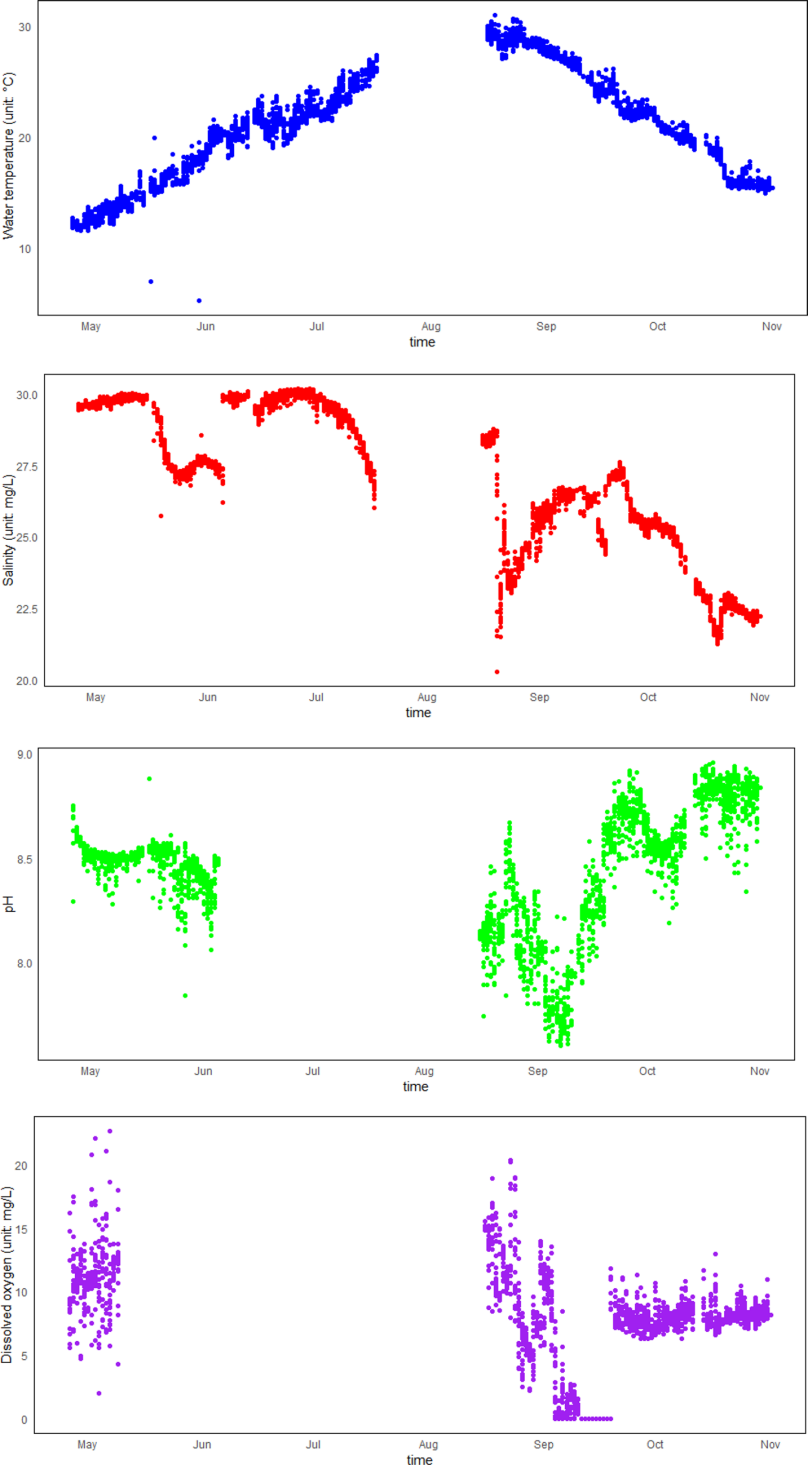

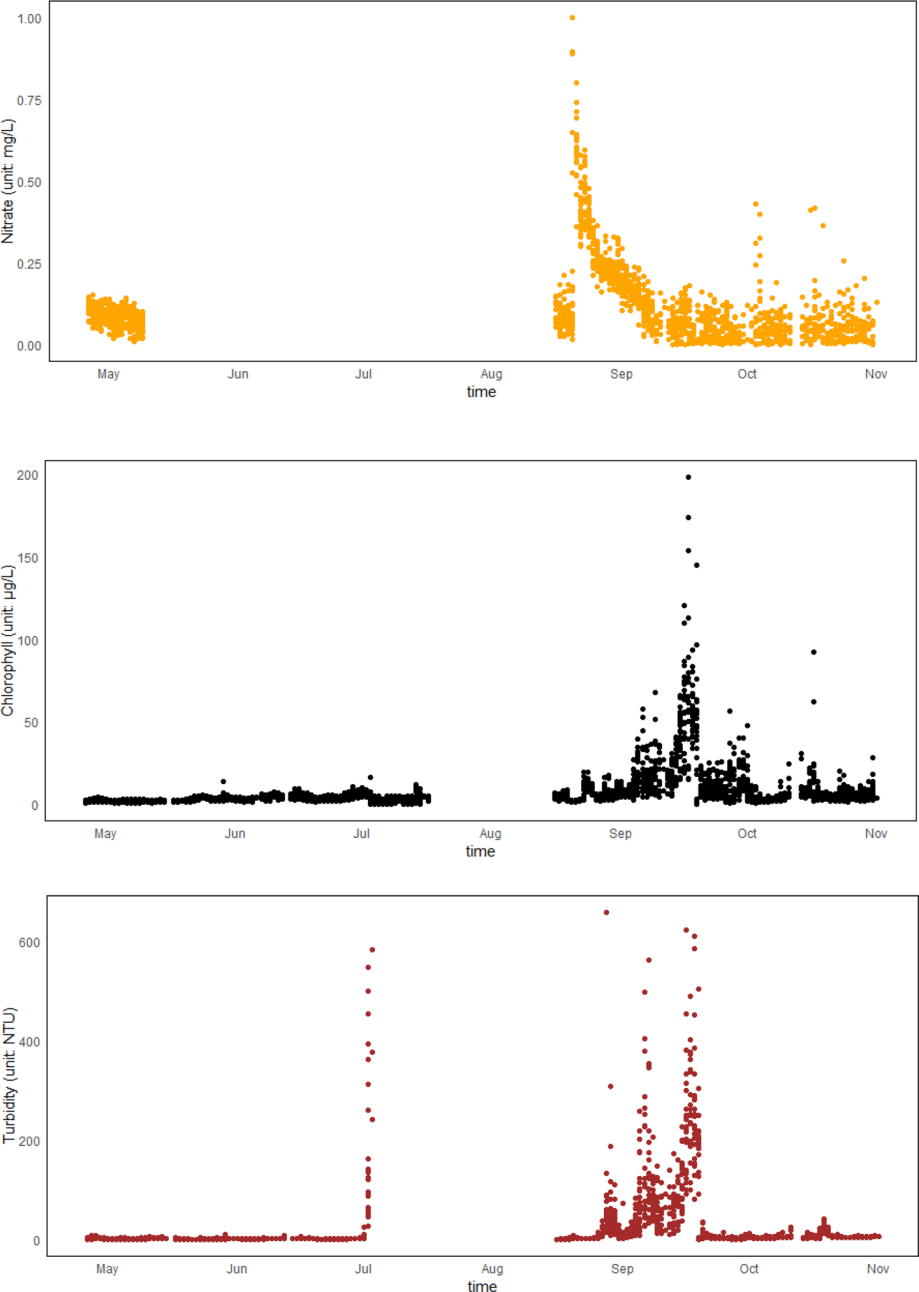
Changes in red tide factors.

Data from the online buoys (Fig. 3) show a water temperature range of 5.27 to 30.98 °C, with an average of 19.25 °C, exhibiting a general trend of first rising and then falling. The highest temperature (30.98 °C) was recorded on August 18 and the lowest (5.27 °C) occurred on May 30. Salinity varied from 20.1 to 30.2, with an average of 24.32. The highest salinity was observed on June 16, displaying a two-phase pattern over time: higher values from April to July, with an average of 29.16, and a decrease to an average of 24.33 from August to October. The pH levels fluctuated from 7.60 to 8.96, with an average of 8.700, showing a trend of first decreasing and then increasing. The minimum pH (7.60) was recorded on September 7, while the maximum (8.96) was on October 9. The DO concentration ranged from 0.00 to 22.68 mg/L, averaging 7.12 mg/L. There was a substantial variation from April to September, but by October, the fluctuation range narrowed to 6.29–12.97 mg/L. Nitrate concentrations ranged from 0.0001 to 1.0019 mg/L, with an average of 0.13962 mg/L, remaining below 0.25 mg/L for most of the monitoring period, except during specific intervals from August 20 to September 1, October 3 to 4, and October 16 to 17. Turbidity ranged from 1.059 to 659.876 NTU, with an average of 20.2843 NTU. Notably, turbidity increased significantly from August 27 to September 21, with an average of 93.1810 NTU, while other periods exhibited lower turbidity, averaging 3.9559 NTU. Chlorophyll-a concentrations varied from 0.000 to 198.525 μg/L, with an average of 11.3578 μg/L. The concentrations were lower from April to July, with an average of 3.2889 μg/L and higher from August to October, with an average of 11.3531 μg/L, peaking at an average of 23.1194 μg/L from September 15 to October 1.

### Environmental factors and level selection for early warning of red tides

The study results (Table 1) indicate a significant negative correlation between the MARs of chlorophyll-a and nitrate and the incidence of red tides, whereas there was no significant correlation between DO and its range in this respect. When the incidence of red tides exceeded 50%, no red tide events were detected in terms of pH or turbidity indicators. The highest incidence for dissolved oxygen and its range was 50%, whereas the maximum incidence for pH and turbidity was only 30%. At 100% incidence of red tides, chlorophyll-a exhibited an exceptionally high MAR of 98.08%, corresponding to concentrations of ≥ 198.525 μg/L. Monitoring data showed that chlorophyll-a concentration above this level was recorded only on one day, and satellite observations indicated the occurrence of a red tide on that day. However, no red tide event was detected at this probability level in terms of other indicators. When the incidence dropped to 90%, 80%, and 70%, all factors had no corresponding indicator concentrations. When the incidence dropped to 60%, the MAR of chlorophyll-a remained at a high level of 96.15%, with a corresponding concentration range of ≥ 120.653 μg/L. The MAR of nitrate was relatively low at 58.54%, with a corresponding concentration range of ≥ 0.1897 mg/L. When the incidence further dropped to 50%, the MAR of DO range was the lowest, reaching 0.00%, while the MAR of DO was the highest at 82.98%, with both having a concentration range ≥ 0 mg/L. At this point, the MARs of chlorophyll-a and nitrate were slightly different, at 42.31% and 48.78%, respectively, with concentration ranges of ≥ 9.452 μg/L and 0.1516 mg/L, respectively. (Note: “-” indicates that no concentration range or MAR was available under the corresponding incidence.)

**Table 1:** Incidence and MARs Corresponding to the Indicators.

Incidence (%)	Chlorophyll		DO		DO Range		Nitrate	
	Range (μg/L)	MAR	Range (mg/L)	MAR	Range (mg/L)	MAR	Range (mg/L)	MAR
100	198.525	98.08%	–	–	–	–	–	–
90	–	–	–	–	–	–	–	–
80	–	–	–	–	–	–	–	–
70	–	–	–	–	–	–	–	–
60	120.653	96.15%	–	–	–	–	0.1897	58.54%
50	9.452	86.53%	0.00	82.98%	0.00	0.00%	0.1516	48.78%

## Discussion

Located in the Bohai Sea region of China, Qinhuangdao occupies a mid-latitude zone characterized by distinct seasons and concentrated precipitation during the summer and autumn^23^. In this study, temperature changes generally exhibited a dynamic pattern of initial increase followed by decrease, while the salinity remained within a specific range, experiencing a notable decline during autumn. This phenomenon may be related to factors such as increased rainfall in the sea area or expanded surface runoff, which concurrently inputs nutrients and causes nitrate concentration fluctuations^24^. The study demonstrates a positive correlation between red tide occurrence and both chlorophyll-a concentration and DO content, while the DO range, pH, and turbidity of the sea area showed a negative correlation with red tide events. No significant correlation was observed between the occurrence of red tides and other environmental indicators. Related research on Noctiluca scintillans red tides in Zhejiang coastal sea area^7^ reported positive correlations among chlorophyll-a, DO, and pH, which is consistent with our observed chlorophyll-a/DO relationship though the pH correlation was not confirmed in this study. Additionally, researchers have identified significant differences in DO measurements between the red tide occurrence period and its non-occurrence period^25^, with a larger DO range during red tide occurrence and decreasing nitrate concentrations accompanying red tides, which aligns with our findings of significant negative correlations between red tide formation and both the DO range and nitrate concentrations.

The phenomenon of red tides has caused significant economic losses and ecological damage^20^. The establishment of an early warning system plays a crucial role in mitigating their negative impacts, making the selection of appropriate early warning parameters essential for the system’s effectiveness. This study identified chlorophyll-a, DO, and its range as key early warning indicators. At a 60% incidence, nitrate demonstrated the lowest MAR of 58.54 to 78.05%, with an early warning range of 0.1897 to 0.3261 mg/L. When the incidence decreased to 50%, the DO exhibited the minimum MAR ranging from 0.00 to 4.25%, with an early warning range of 11.09 to 11.68 mg/L. Red tide occurrence showed significant correlation with chlorophyll-a^7,25^, where 10 μg/L may be viewed as an early warning indicator for red tide occurrence, which is consistent with this study’s finding of a chlorophyll concentration of 9.452 µg/L as an early warning indicator, though with significantly higher MAR compared to DO. Previous research indicates the DO range outperforms direct DO measurement as an early warning indicator^7^. In this study, DO and its range were used as warning indicators: at 50% incidence, the DO range achieved 0.00% MAR versus 82.98% for direct DO measurement, further validating the reliability of this approach.

The results of this study demonstrate that during the field survey conducted on August 14 based on satellite remote sensing data, the density of *Ceratium furca* reached 662,000 cells/L, significantly exceeding the 100,000 cells/L threshold specified in the National Red Tide Monitoring Program. This validates the determination of red tide occurrences based on satellite data and the statistical indicators derived from these events. As the incidence decreased from 100 to 60%, all indicators exhibited corresponding ranges within the 90%, 80%, and 70% occurrence intervals. Taking chlorophyll-a an example of an early warning indicator for red tide events, statistical data indicates a disproportionate increase in monitored versus actual red tide days: when actual red tide duration increased by 1 day, the number of monitored event days increased by 2; a 2-day actual increase corresponded to 6 detected days; and a 3-day actual increase resulted in 7 detected days, with number of monitored event days increased by 7 days. In all these scenarios, the monitored red tide event days exceeded the actual occurrence days by a factor of two or more. This disproportionate increase in monitored days has led to a rapid decline in the red tide occurrence rate.

During red tide events, chlorophyll concentrations exhibited spatial heterogeneity influenced by the speed and direction of ocean currents, resulting in irregular red tide distribution patterns^26^. When buoys are positioned at different locations of a red tide, their effectiveness in monitoring varies^27^: When buoys are set outside the red tide-affected area, the monitored early warning indicators will be unaffected, making it impossible to issue a red tide early warning. When situated at the periphery, they show insignificant parameter fluctuations, leading to delays in early warning information. Conversely, when buoys are deployed within the center of the red tide area, buoys promptly capture environmental parameter variations, allowing for rapid generation of red tide early warnings and timely responses. Furthermore, even if the spread of the red tide appears in a band-like expansion, even if a buoy is near the red tide area, it may still fail to detect data fluctuations. To ensure comprehensive monitoring, accurate assessment, and real-time tracking of critical red tide indicators, it is recommended to deploy multiple monitoring buoys in areas prone to red tide occurrences.

## Conclusion

During the study, a total of 16 red tide events were recorded, with a cumulative duration of 55 days and the total affected area of 10,047 km^2^. The observed temperature varied from 12.23 to 30.59 °C and salinity from 23.04 to 30.21. Statistical analysis results indicate that the nitrate concentration and DO range of the sea area concerned are more reliable as early warning indicators: when the nitrate concentration reached 0.1897 mg/L, the red tide forecast accuracy reached 60% with an MAR of 58.54%; when the DO range was 0.00 mg/L, the forecast accuracy of red tides was 50%, with a 0.00% MAR.

## Data Availability

Due to the sensitive nature of the issues involved, the data on which the findings of this study are based have not been made public. However, upon reasonable request, the corresponding author can provide these data.

## References

[1] Bi, F. et al. Progress in red tide forecasting technology and its application prospects in Qinhuangdao coastal waters. *Mar. Forecasts* **5**, 1–22 (2024).

[2] Zhan, H. L. & Rao, X. Z. Research progress on the harm, causes and prevention of red tides. *Biol. Teach.* **46**(7), 66–68 (2021).

[3] Yu, Z. M. & Chen, N. S. Global trends and research hotspots of red tides. *Oceanol. Limnol. Sin.* **50**(3), 474–486 (2019).

[4] Yu, R. C. et al. Current status and prospects of research on harmful algal blooms in China’s coastal waters. *Oceanologia et Limnologia Sinica* **51**(4), 768–788 (2020).

[5] Xu, H. L. et al. Analysis of marine red tide disaster characteristics based on time series. *Mar. Sci. Bull.* **33**(4), 469–474 (2014).

[6] Chen, X. Y. & Liu, B. L. Application of marine online monitoring buoys in red tide monitoring. *J. Trop. Oceanogr.* **37**(5), 20–24 (2018).

[7] Sun, X. X. Early warning and decision-making services for red tides using buoy and satellite data. Master’s thesis, Zhejiang University (2017).

[8] Zhao, C. J. et al. Analysis of *Karenia mikimotoi* red tide process and environmental factor variation characteristics based on online water quality buoy monitoring. *J. Trop. Oceanogr.* **39**(2), 88–97 (2020).

[9] Harding, J. L. W. et al. Variable climatic conditions dominate recent phytoplankton dynamics in Chesapeake Bay. *Sci. Rep.* **6**, 23773 (2016). 27026279 10.1038/srep23773 PMC4824454

[10] Yang, W. et al. Research on artificial intelligence red tide early warning in Qinhuangdao Bay. *Mar. Inf. Technol. Appl.* **37**(1), 56–64 (2022).

[11] Li, X. Study on the influence of sea surface temperature on red tides in Huangqi, Lianjiang. *Mar. Forecasts* **38**(3), 95–103 (2021).

[12] Shi, X. Y. et al. Preliminary study on hydrochemical characteristics of the Taiwan Warm current in summer and its influence on the high frequency area of red tides in the East China Sea. *Oceanologia et Limnologia Sinica* **44**(5), 1208–1215 (2013).

[13] Zhang, C. S. Characteristics of red tide evolution process and its nutrient effect in the Changjiang Estuary and adjacent waters. PhD dissertation, Ocean University of China (2008).

[14] Zhang, J. et al. Relationship between red tides and environmental factors in coastal waters of the East China Sea. *J. Guangdong Ocean Univ.* **39**(1), 66–70 (2019).

[15] Guo, W. Sedimentary records analysis of hypoxia in the East China Sea red tide area. Master’s thesis, Institute of Oceanology, Chinese Academy of Sciences (2013).

[16] Wang, D. et al. Relationship between red tide organisms and environmental factors in Beidaihe, Qinhuangdao. *Mar. Forecasts* **30**(5), 1–7 (2013).

[17] Zhang, X. L. & Yan, C. Y. Analysis of meteorological conditions for red tide occurrence in Qinhuangdao coastal waters. *J. Anhui Agric. Sci.* **45**(35), 186–189 (2017).

[18] Han, X. R. et al. Distribution characteristics of nutrients and their relationship with red tide occurrence in the coastal area of the East China Sea. *Chin. J. Appl. Ecol.* **14**(7), 1097–1101 (2003). 14587329

[19] Qiu, Y. W. et al. Temporal variation rates of phytoplankton and nutrients during red tide processes. *Chin. J. Appl. Ecol.* **14**(7), 1127–1130 (2003). 14587335

[20] Yang, J. et al. Analysis of influencing factors of chlorophyll-a in the red tide monitoring area of Bohai Bay in 2006. *Oceanologia et Limnologia Sinica* **43**(6), 1023–1029 (2012).

[21] Dou, Y. et al. Occurrence patterns and influencing factors of red tides in southern coastal waters of China from 2000 to 2013. *J. Hydroecol.* **36**(3), 31–37 (2015).

[22] Dou, Y. et al. Occurrence patterns and influencing factors of red tides in the Bohai Sea from 2000 to 2016. *J. Hydroecol.* **41**(6), 141–148 (2020).

[23] Zhang, Y. J. et al. Characteristics analysis and countermeasures of red tide events in Qinhuangdao coastal waters from 1990 to 2023. *J. Hebei Univ. Environ. Eng.* **6**, 1–7 (2024).

[24] Wu, T. et al. Long-term variation analysis of nutrients in Qinhuangdao coastal waters based on statistical methods. *Mar. Environ. Sci.* **34**(4), 499–502 (2015).

[25] Li, F. et al. Real-time automated red tide early warning based on machine learning using buoy data. *Environ. Monit. China* **39**(4), 196–205 (2023).

[26] Liu, H. J. et al. Seasonal variations of phytoplankton chlorophyll a and primary production and their influencing factors in the Pearl River Estuary. *J. Trop. Oceanogr.* **36**(1), 81–91 (2017).

[27] He, E. Y. et al. Research on short-term forecasting method of Chl-a concentration based on serial deep neural network. *Mar. Forecasts* **38**(4), 1–10 (2021).

